# Association of knee pain and different definitions of knee osteoarthritis with health-related quality of life: a population-based cohort study in southern Sweden

**DOI:** 10.1186/s12955-016-0525-4

**Published:** 2016-08-26

**Authors:** Aliasghar A. Kiadaliri, Carl Johan Lamm, Maria Gerhardsson de Verdier, Gunnar Engström, Aleksandra Turkiewicz, L. Stefan Lohmander, Martin Englund

**Affiliations:** 1 Clinical Epidemiology Unit, Orthopaedics, Department of Clinical Sciences-Lund, Lund University, Lund, Sweden; 2 Health Services Management Research Center, Institute for Futures Studies in Health, Kerman University of Medical Sciences, Kerman, Iran; 3 Astra Zeneca R&D, Mölndal, Sweden; 4 Astra Zeneca R&D, Gothenburg, Sweden; 5 Cardiovascular Epidemiology, Department of Clinical Sciences, Lund University, Malmö, Sweden; 6 Research Unit for Musculoskeletal Function and Physiotherapy, University of Southern Denmark, Odense, Denmark; 7 Department of Orthopedics and Traumatology, University of Southern Denmark, Odense, Denmark; 8 Epidemiology and Register Centre South, Skåne University Hospital, Lund, Sweden; 9 Clinical Epidemiology Research and Training Unit, Boston University School of Medicine, Boston, MA USA; 10 Skånes University Hospital, Clinical Epidemiology Unit, Klinikgatan 22, SE-221 85 Lund, Sweden

**Keywords:** EQ-5D-3L, Knee osteoarthritis, Knee pain, KOOS, Quality of life, Sweden

## Abstract

**Background:**

While the impact of knee pain and knee osteoarthritis (OA) on health-related quality of life (HRQoL) has been investigated in the literature, there is a lack of knowledge on the impact of different definitions of OA on HRQoL. The main aim of this study was to measure and compare the impact of knee OA and its different definitions on HRQoL in the general population.

**Methods:**

A random sample of 1300 participants from Malmö, Sweden with pain in one or both knees in the past 12 months with duration ≥4 weeks and 650 participants without were invited to clinical and radiographic knee examination. A total of 1527 individuals with a mean (SD) age 69.4 (7.2) participated and responded to both generic (EQ-5D-3L) and disease-specific (the Knee injury and Osteoarthritis Outcome Score) questionnaires. Knee pain was defined as pain during the last month during most of the days. Knee OA was defined radiographically (equivalent to Kellgren and Lawrence grade ≥2) and clinically according to the American College of Rheumatology (ACR) criteria.

**Results:**

Of participants with either knee pain or knee OA or both, 7 % reported no problem for the EQ-5D-3L attributes. The corresponding proportion among references (neither knee pain nor OA) was 42 %. The participants with knee pain and OA had all HRQoL measures lower compared to those with knee pain but *no* OA. The ACR clinical definition of knee OA was associated with lower HRQoL than the definition based on radiographic knee OA (adjusted difference −0.08 in UK EQ-5D-3L index score).

**Conclusions:**

Applying different definitions of knee OA result in different levels of HRQoL and this is mainly explained by the knee pain experience. These differences may lead to discrepant conclusions from cost-utility analyses.

## Background

The prevalence of knee osteoarthritis (OA) rapidly increases with age [1, 2]. Thus, it is expected that the number of people with knee OA will increase in the future due to the steadily aging population and increasing prevalence of obesity [3, 4]. The Global Burden of Disease (GBD) 2010 study ranked hip and knee OA as the 11^th^ leading cause of years lived with disability (YLD) and the 38^th^ highest contributor to disability-adjusted life years (DALYs) among 291 conditions [5]. The share of total DALYs attributed to hip and knee OA increased from 0.49 % in 1990 to 0.69 % in 2010 (an estimated 64 % increase) [5].

OA is associated with substantial deteriorations in health-related quality of life (HRQoL) [6]. A recent study in the UK ranked OA as the 3^rd^ greatest contributor to loss of HRQoL among eleven long-standing health conditions [7]. HRQoL constitutes a subjective and multidimensional concept that includes the physical, psychological, and social functioning related to a health condition or therapy [8]. HRQoL can be used as a criterion to predict future health care consumption among people with OA [9]. Therefore, measuring HRQoL is important not only to quantify the effects of disease and its treatments, but also to aid informed decision-making in allocation of often limited healthcare resources.

The relatively weak association between clinical symptoms of OA and radiographic evidence of the disease has contributed to the existence of several definitions of OA for study purposes. The multiple definitions represent a major challenge in studies on OA [10]. A radiographic definition of OA (most commonly using Kellgrene-Lawrence scale [11] with the cut-off of grade 2 or worse) and the clinical definition proposed by the American College of Rheumatology (ACR) [12] are two most commonly used definitions in the literature. While the impact of knee pain and knee OA on HRQoL has been investigated in the literature [13–15], there is a lack of knowledge on the impact of *different* definitions of OA on HRQoL. If such differences exist, the different OA definitions used in health economic evaluations may potentially yield different results and conclusions. Thus, in the present study our main aim was to evaluate and compare the impact of these two definitions of knee OA on HRQoL. These were based on patient-reported knee pain, and/or radiographically evident knee OA, as well as clinically defined knee OA. We related our findings to knee-healthy reference participants drawn from the same source population.

## Method

### Setting and participants

The Malmö OA study (MOA) originated from the Malmö Diet and Cancer Study (MDCS) cohort established between 1991 and 1996 [16]. All men aged 45–73 years and women aged 44–74 years at the time of enrolment living in the city of Malmö were invited to participate in the MDCS (*n* = 74 138). A cohort of 28 098 had completed baseline examinations [17]. In the year 2007 a postal questionnaire about knee pain was sent to a 10 000 random sample from the MDCS who completed the MDCS baseline examination and were still alive and resident in the Malmö area. Respondents answered a question about whether they had knee pain during the previous 12 months and its duration (<1 week, 1–4 weeks, 1–3 months, >3 months). In the second stage, a random sample of 1300 participants with pain in one or both knees in the past 12 months and duration of at least 4 weeks (group A) and 650 participants without knee pain or with knee pain for shorter duration (group B) were invited to a clinical visit and radiographic examination [16, 18]. Of these, 1028 (79 %) people from group A and 499 (77 %) people from group B participated and were included in the present study.

### Radiographic evaluation and knee OA definitions

Both knees were radiographed in a weight-bearing and semi-flexed position (knees in 10–15° of flexion) using a posterior-anterior beam direction (film focus distance 110 cm, 60 kV and 10 mA) with the aid of fluoroscopy to optimally align the tibia plateau. An independent senior radiologist specialized in musculoskeletal conditions who was blinded to the clinical data assessed *joint space narrowing* and *osteophytes* according to the atlas from the Osteoarthritis Research Society International [16, 19]. We classified a knee as having *radiographic knee OA* if one or more of the following criteria were fulfilled in either the medial, lateral tibiofemoral compartment or patellofemoral compartment: joint space narrowing grade 2 or worse, the sum of marginal osteophyte grades in the same compartment 2 or worse, joint space narrowing grade 1 and osteophyte grade 1 in the same compartment (approximating Kellgren and Lawrence (KL) grade 2 or worse) [20].

All participants answered the 5 questions included in the ACR clinical criteria for knee OA. We classified a participant as having *clinical OA* if fulfilling ACR clinical criteria according to the recursive partitioning method [12]. Five items are included in the ACR criteria (28): (a) knee pain for most days of the prior month, (b) crepitus on active joint motion, (c) morning stiffness of duration < 30 min, (d) age ≥ 38 years, and (e) bony enlargement of the knee on examination. Knee OA was present if items “a”, “b”, “c”, “d” or items “a”, “b”, “e” or items “a” and “e” were present [12]. We used the ACR clinical criteria “a” (i.e. knee pain for most days of the prior month) to define knee pain in our study. It should be noted that as this definition was different from the definition of knee pain in the first stage and also due to gap between the first and second stages of the study, people who defined as having knee pain in the first stage could have been classified as not having knee pain and vice versa.

We classified participants into four exposure groups based on their clinical and radiographic knee status: 1) *reference group* having neither knee pain nor radiographic or clinically-defined knee OA (*n* = 744), 2) *knee pain without OA*, i.e., participants with knee pain but without radiographic or clinically-defined knee OA (*n* = 169), 3) *knee pain with OA*, i.e., participants with knee pain having either radiographic knee OA or clinically-defined knee OA (*n* = 402), and 4) *radiographic knee OA but no knee pain*, i.e. participants without knee pain but fulfilling the radiographic defined knee OA (*n* = 186). A total of 26 participants with missing value on knee OA status were excluded. Additionally, to assess the impact of different definition of knee OA on HRQoL, the participants with knee OA (i.e., groups 3 & 4) were collapsed, then classified into three subgroups: those fulfilling the criteria for a) radiographic knee OA (*n* = 282), b) clinical knee OA (*n* = 157), and c) both clinical and radiographic knee OA (*n* = 149).

### Health-related quality of life measurement

The EQ-5D-3L is a generic multi-attribute instrument to elicit health-related preferences. The EQ-5D-3L covers five attributes: mobility, self-care, usual activities, pain/discomfort, and anxiety/depression. Each attribute has three levels: no problems, moderate problems, and severe problems, resulting in 243 (3^5^) possible health states [21]. The responses to these attributes are weighted based on the preferences elicited from a general population/patients sample to calculate an index score. We used the UK [22] and recently developed Swedish [23] sets of preferences to calculate the index score. The UK EQ-5D-3L scores range between −0.594 and 1 (full health), while the Swedish scores range between 0.340 and 0.969.

The Knee injury and Osteoarthritis Outcome Score (KOOS) is a validated knee specific instrument [24, 25]. The KOOS is a 42-item self-administered questionnaire covering 5 subscales: pain, other symptoms, function in daily living (ADL), function in sport and recreation (Sport/Rec) and knee related quality of life (QoL). All items have five possible answer options scored from 0 (no problems) to 4 (extreme problems). A normalized score (100 indicating no symptoms and 0 indicating extreme symptoms) is calculated for each subscale. As our participants were an elderly population, we included a sixth answer option (not applicable) into the case report form for the subscale Sport/Rec. If the box “not applicable” was marked, the item was treated as missing data. Since a large number of participants selected this option, we decided not to include this subscale in our analysis.

### Explanatory variables (confounders)

To avoid potential confounder bias, the following explanatory variables were included in our regression analysis: sex, age, body mass index (BMI), comorbidity, years of education, employment, and smoking. Age and BMI were included as continuous variables. Comorbidity was defined as presence of self-reported doctor’s diagnosis of one or more of the following comorbidities: back problems, other joint problems (except knees), asthma/lung disease, hypertension, heart disease, stroke, leg artery disease, neurological disease, diabetes, cancer, gastric ulcer, renal disease, anemia/blood disease, eye disease/visual impairment, balance disorder, depression, and other psychiatric disorder. Three groups were defined: no comorbidity, single comorbidity, and multiple comorbidities. Years of education were categorized in three groups: ≤9 years, 9–12 years, and >12 years. Employment was defined in three categories: employed, unemployed, and retired. We grouped smoking in three groups as: never, current smoker, and ex-smoker.

### Statistical analysis

We applied weighting to account for a possible selection bias that might arise from non-responses in the first or second part in the MOA study [26]. A logistic regression model with sex, age on 1 January 2007, education, smoking, and BMI as covariates was used to estimate the probability of response in the first stage of survey. Similar logistic regression models including knee pain during last 12 months as an additional covariate were applied to estimate the probability of participation and attendance at the clinical examination. The sampling weights (the reciprocal of the sampling probability for those with and without knee pain ≥ 4 weeks duration) were multiplied by the weights for response, participation and attendance to construct the final weights used in analyses.

We present continuous variables using means and standard deviations. The mean differences between groups were tested using one-way analysis of variance (ANOVA) and Bonferroni post-hoc tests. Proportions were compared using Pearson Chi-2 test. Adjusted analysis was conducted using Ordinary least squares regression with robust standard errors. Due to the skewed distribution of the EQ-5D-3L data, several different methods have been applied to these data in the literature [27, 28], but ordinary least squares (OLS) regression is considered as a simple and valid approach [29]. The linearity of the continuous variables was checked using design variables and residual plots and non-linearity was modelled using fractional polynomial. The possible reasonable linear and non-linear interactions were also checked using the same approach [30]. Potential confounders (age, sex, body mass index, comorbidity, years of education, employment, and smoking) were included in the models regardless of their significance level. STATA version 13 (StataCorp LP, College Station, TX, USA) was used for statistical analysis.

## Results

The mean (SD) age and BMI of the participants included was 69.4 (7.2) and 27.7 (4.9), respectively, and 63.8 % were women (Table 1). Except sex and smoking, there were no statistically significant differences in other explanatory variables across the study groups.

**Table 1:** Characteristics of participants included in the Malmö osteoarthritis study (MOA)

	Reference group (*n* = 744)	Knee pain *without* knee OA (*n* = 169)	Knee pain *with* knee OA (*n* = 402)	Radiographic knee OA *without* pain (*n* = 186)
Women, %	63.0	70.4	63.7	61.3
Age, years (SD)	68.7 (7.2)	68.4 (7.3)	70.1 (7.2)	71.5 (6.7)
BMI (SD)	26.7 (4.3)	27.8 (4.8)	28.9 (5.5)	28.8 (5.1)
Comorbidity, %				
Single	32.5	26.6	23.1	28.0
Multiple	46.1	59.2	64.9	53.2
Years of education, %				
≤ 9 years	35.0	30.3	43.3	34.4
> 9 years & ≤ 12 years	38.3	43.2	34.7	44.8
>12 years	26.7	26.5	22.0	20.8
Smoking, %				
Current smoker	14.1	12.7	12.3	11.8
Ex-smoker	45.1	46.4	46.0	42.5
Employment, %				
Employed	25.3	23.7	14.7	15.6
Retired	72.2	72.8	80.9	81.7

The mean UK EQ-5D-3L index scores were 0.69 (95 % CI 0.67 to 0.70) and 0.85 (95 % CI 0.83 to 0.86) in non-reference and reference groups, respectively. Across non-reference groups, the participants with radiographic knee OA without knee pain had generally less problems on the EQ-5D-3L dimensions and reported statistically significantly higher HRQoL scores compared with the participants who had knee pain (Table 2). Among the participants with knee pain, the participants with both knee pain and knee OA reported lower HRQoL scores.

**Table 2:** Percentage (95 % CI) of participants with moderate/severe problems in the EQ-5D-3L attributes, mean (95 % CI) EQ-5D-3L index and KOOS scores in the full sample

	Reference group (*n* = 744)	Knee pain *without* knee OA (*n* = 169)	Knee pain *with* knee OA (*n* = 402)	Radiographic knee OA without knee pain (*n* = 186)
EQ-5D-3L dimension				
Mobility	11.5 (8.9 to 14.8)	32.6 (23.1 to 43.8)^a^	52.2 (44.8 to 59.5)^ab^	34.7 (25.4 to 45.5)^ac^
Self-care	0.2 (0.1 to 0.5)	2.7 (1.3 to 5.6)^a^	7.3 (3.5 to 14.5)^a^	3.6 (1.0 to 12.3)^a^
Usual activities	5.6 (3.9 to 8.0)	21.9 (14.3 to 32.0)^a^	24.8 (20.1 to 30.3)^a^	10.0 (5.1 to 18.7)^bc^
Pain/discomfort	35.9 (31.8 to 40.3)	88.1 (76.1 to 94.5)^a^	96.7 (92.1 to 98.7)^ab^	70.4 (60.1 to 78.9)^abc^
Anxiety/depression	17.5 (14.3 to 21.1)	30.2 (20.9 to 41.3)^a^	32.2 (25.9 to 39.4)^a^	21.4 (13.9 to 31.4)
No problem at any dimension (full health)	54.2 (50.0 to 58.6)	7.7 (2.9 to 19.0)^a^	3.1 (1.2 to 7.8)^a^	22.6 (15.2 to 32.2)^abc^
Mean UK EQ-5D-3L index score	0.89 (0.87 to 0.90)	0.71 (0.67 to 0.75)^a^	0.67 (0.64 to 0.69)^ab^	0.77 (0.72 to 0.82)^abc^
Mean Swedish EQ-5D-3L index score	0.93 (0.93 to 0.94)	0.87 (0.85 to 0.89)^a^	0.84 (0.83 to 0.85)^ab^	0.89 (0.87 to 0.91)^abc^
KOOS subscale				
Pain	92.8 (91.8 to 93.8)	68.9 (64.3 to 73.4)^a^	55.4 (52.9 to 57.8)^ab^	81.4 (78.2 to 84.6)^abc^
Symptoms	66.1 (65.4 to 66.8)	56.7 (54.4 to 58.9)^a^	48.2 (46.5 to 50.0)^ab^	61.6 (59.5 to 63.8)^abc^
ADL	91.3 (90.1 to 92.6)	70.3 (65.6 to 74.9)^a^	56.2 (53.1 to 59.3)^ab^	80.1 (76.4 to 83.9)^abc^
QoL	86.4 (84.9 to 87.9)	56.9 (51.6 to 62.1)^a^	40.1 (37.4 to 42.9)^ab^	66.4 (61.9 to 70.8)^abc^

*ADL* function in daily living, *QoL* knee-related quality of life

^a^ *P* < 0.05 compared with reference group

^b^ *P* < 0.05 compared with knee pain without knee OA

^c^ *P* < 0.05 compared with knee pain with knee OA

Among the participants with knee OA, those with radiographic knee OA generally experienced less problems in the EQ-5D-3L dimensions and reported statistically significantly higher HRQoL scores than the participants fulfilled clinical knee OA definition (Table 3). The participants with both radiographic and clinical knee OA reported lower scores on three KOOS subscales compared with the participants with clinically defined knee OA.

**Table 3:** Percentage (95 % CI) of participants with moderate/severe problems in the EQ-5D-3L attributes, mean (95 % CI) EQ-5D-3L index and KOOS scores among participant with knee osteoarthritis (OA)

	Radiographic knee OA (*n* = 282)	Clinical knee OA (*n* = 157)	Clinical and radiographic knee OA (*n* = 149)
EQ-5D-3L dimension			
Mobility	40.5 (32.1 to 49.5)	38.9 (29.3 to 49.5)	59.7 (48.7 to 69.8)^ab^
Self-care	5.5 (2.1 to 13.6)	5.0 (2.7 to 9.4)	5.3 (2.6 to 10.5)
Usual activities	12.2 (7.6 to 18.9)	27.4 (20.2 to 36.0)^a^	26.5 (19.3 to 35.2)^a^
Pain/discomfort	76.3 (67.8 to 83.2)	98.6 (95.6 to 99.5)^a^	94.4 (82.2 to 98.4)^a^
Anxiety/depression	23.4 (16.7 to 31.8)	36.0 (25.5 to 48.1)	29.7 (22.0 to 38.7)
No problem at any dimension (full health)	18.3 (12.4 to 26.1)	0.9 (0.2 to 3.5)^a^	5.6 (1.6 to 17.8)^ab^
Mean UK EQ-5D-3L index score	0.75 (0.71 to 0.79)	0.67 (0.63 to 0.71)^a^	0.66 (0.61 to 0.70)^a^
Mean Swedish EQ-5D-3L index score	0.88 (0.87 to 0.90)	0.84 (0.82 to 0.86)^a^	0.84 (0.81 to 0.86)^a^
KOOS subscale			
Pain	75.6 (72.4 to 78.9)	58.6 (54.8 to 62.4)^a^	52.0 (47.5 to 56.6)^ab^
Symptoms	59.5 (57.6 to 61.4)	48.2 (45.6 to 50.7)^a^	45.0 (42.4 to 47.6)^a^
ADL	74.6 (70.8 to 78.4)	59.5 (54.4 to 64.6)^a^	53.6 (49.2 to 58.1)^ab^
QoL	60.5 (56.5 to 64.5)	44.2 (40.1 to 48.3)^a^	35.9 (30.3 to 41.5)^ab^

*ADL* function in daily living, *QoL* knee-related quality of life

^a^ *P* < 0.05 compared with radiographic knee OA

^b^ *P* < 0.05 compared with clinical knee OA

Controlling for potential confounders in the regression analysis did not alter our unadjusted findings in the full sample (Table 4). Generally the outcome measures were ranked in following order: reference group > radiographic knee OA without knee pain > knee pain without knee OA > knee pain with knee OA. Among the participants with knee OA, while the participants with radiographic knee OA reported higher scores on all outcome measures, the difference between two other groups (i.e., clinical knee OA only and clinical and radiographic knee OA) was statistically significant only on the KOOS-Pain subscale.

**Table 4:** The effects of knee osteoarthritis (OA) on health-related quality of life: results from regression analysis

	UK EQ-5D-3L index score	Swedish EQ-5D-3L index score	Pain (KOOS)	Symptoms (KOOS)	ADL (KOOS)	QoL (KOOS)
Full sample^a^						
Reference group	0.00 (ref)					
Knee pain *without* knee OA	−0.15***	−0.06***	−22.69***	−8.78***	−19.67***	−27.41***
Knee pain *with* knee OA	−0.19***	−0.08***	−35.14***	−16.92***	−31.94***	−43.48***
Radiographic knee OA *without* pain	−0.09***	−0.03**	−10.04***	−4.27***	−8.72***	−18.47***
Participants with knee OA^a^						
Radiographic knee OA	0.00 (ref)					
Clinical knee OA	−0.08***	−0.04***	−17.92***	−10.91***	−17.40***	−18.40***
Clinical and radiographic knee OA	−0.10**	−0.05***	−23.79***	−14.08***	−22.50***	−24.42***

*ADL* function in daily living, *QoL* Knee-related quality of life

***,** *p* < 0.001, and *p* < 0.01 significance level compared to the reference group

^a^Both models were additionally adjusted for age, sex, body mass index, comorbidity, years of education, employment, and smoking

## Discussion

We measured and compared the impact of knee pain and knee OA on HRQoL, using both generic and disease-specific scales, in a large population-based cohort from southern Sweden. First, our results confirmed the expected notion that participants with either knee pain or knee OA (either clinically or radiographically defined) reported lower HRQoL scores than reference participants free of such knee pain and with no knee OA. We also found that knee pain without knee OA had more profound negative impact on HRQoL than radiographic knee OA without knee pain. Moreover, among participants with knee OA (either radiographically *or* clinically defined), those with *clinical* knee OA according to the ACR clinical definition reported lower scores of HRQoL than those who had radiographic knee OA not fulfilling the ACR clinical criteria.

The effect of different OA definitions in the study of prevalence and incidence of OA is well documented, where it was reported that radiographic definition of OA resulted in the highest prevalence estimates [10]. The effect of different OA definitions on HRQoL is however much less well investigated, which may have important implications for the interpretation of findings from health economic evaluations. Our results suggest that the knee OA definition based on the ACR clinical criteria was associated with lower HRQoL than the definition based on radiographic knee OA. The presence of knee pain in the ACR clinical definition is a possible explanation for this finding. Interestingly, in our full sample, people with knee pain had lower HRQoL compared with people with radiographic features of knee OA without knee pain. In our sample, only 96 (22 %) of participants in the radiographic knee OA group experienced pain in most days during last month and interestingly the EQ-5D-3L index score for these 96 participants (0.66) was equal to participants in the clinical knee OA group (0.66).

Our finding of differences in HRQoL measures between persons fulfilling different definitions of knee OA (i.e., radiographic or clinical ACR criteria) has an important implication for cost-utility analyses. The difference in the mean HRQoL according to knee OA definition will result in different estimates of quality-adjusted life years (QALYs) which might yield discrepant and potentially conflicting conclusions from these analyses. For example, in our study, if a hypothetical intervention improved HRQoL of participants with knee OA to the level of the reference group (0.85), then QALYs gained from using clinical knee OA to define participants with knee OA (0.85 minus 0.66) would be 1.9 times higher than applying radiographic signs for definition of knee OA (0.85 minus 0.75). This translates into less favorable cost-effectiveness ratios using radiographic signs to define knee OA. This should be taken into account by policy makers when using cost-utility analysis to make decisions on funding of knee OA interventions.

In line with previous studies [31–33], we found that pain was the most affected dimension in the EQ-5D-3L questionnaires among participants with knee pain and knee OA. In our study the participants suffering from both knee pain and knee OA reported lower scores for all KOOS subscales compared to people with either knee pain or radiographic knee OA only. In addition, among participants with knee OA, radiographic knee OA without pain was associated with higher HRQoL than radiographic knee OA with pain. These findings suggest that the *combination* of knee pain and knee OA results in additional negative impact on HRQoL compared with knee pain or radiographic knee OA alone.

Mean EQ-5D-3L index score among participants with knee pain and knee OA in our study (0.65) was higher than people with knee OA in Singapore (0.49) [31] and UK (0.44) [34] using the UK weights. Differences in severity of knee OA, with a substantial portion of participants in those studies having severe disease and waiting for TKR might be an explanation. However, our EQ-5D-3L score is comparable to values reported among Swedish people waiting for total hip replacement (0.73) [35] and ACL surgery (0.69) [36]. This similarity between Swedish patients with different severity highlights another potential explanation for higher scores among the Swedish patients than patients in other countries, i.e., clinical, environmental, organizational, and cultural differences between patients and countries in these studies.

The results of this study should be interpreted in light of some limitations. The MOA study originated from the MDCS whose participants were shown to have a slightly lower mortality than non-participants [17]. Eleven percent of people with knee pain in the past 12 months and 30 % of participants without knee pain in past 12 month did not agree to participate in our study. If non-participation was associated with both knee pain/knee OA and HRQoL through unmeasured factors in our weighting exercise, then this is a potential source of selection bias and also could limit the generalizability of our findings. Another limitation of the current study is the self-reported nature of some explanatory variables (e.g., self-reported doctor-diagnosed comorbidity and smoking) that might be prone to recall bias or underreporting. The cross-sectional design of our study implies that any causal inference should be avoided.

## Conclusion

The current study showed that participants with knee pain (with and without knee OA) have poorer HRQoL, measured by both generic and disease-specific scales, than the references. The presence of knee OA has additional negative impact on HRQoL above the knee pain alone. Importantly, we found that applying different definitions of knee OA resulted in different levels of HRQoL and this was mainly explained by the knee pain experience. These differences are important to take into account when assessing the impact of knee OA on HRQoL and interpreting the results of cost-utility analyses.
